# Minimal improvement of nurses’ motivational interviewing skills in routine diabetes care one year after training: a cluster randomized trial

**DOI:** 10.1186/1471-2296-14-44

**Published:** 2013-03-28

**Authors:** Renate Jansink, Jozé Braspenning, Miranda Laurant, Ellen Keizer, Glyn Elwyn, Trudy van der Weijden, Richard Grol

**Affiliations:** 1 Scientific Institute for Quality of Healthcare, Radboud University Nijmegen Medical Centre, P.O. Box 9101, Nijmegen, 6500 HB, The Netherlands; 2 Department of Primary Care and Public Health, School of Medicine, Cardiff University, Heath Park CF14 4XN, Wales, UK; 3 Department of General Practice, Caphri School for Primary Care and Public Health, Maastricht University, P.O. Box 616, Maastricht, 6200 MD, The Netherlands

**Keywords:** Type 2 diabetes, Primary care nurses, Motivational interviewing, Lifestyle, General practice

## Abstract

**Background:**

The effectiveness of nurse-led motivational interviewing (MI) in routine diabetes care in general practice is inconclusive. Knowledge about the extent to which nurses apply MI skills and the factors that affect the usage can help to understand the black box of this intervention. The current study compared MI skills of trained versus non-trained general practice nurses in diabetes consultations. The nurses participated in a cluster randomized trial in which a comprehensive program (including MI training) was tested on improving clinical parameters, lifestyle, patients’ readiness to change lifestyle, and quality of life.

**Methods:**

Fifty-eight general practices were randomly assigned to usual care (35 nurses) or the intervention (30 nurses). The ratings of applying 24 MI skills (primary outcome) were based on five consultation recordings per nurse at baseline and 14 months later. Two judges evaluated independently the MI skills and the consultation characteristics time, amount of nurse communication, amount of lifestyle discussion and patients’ readiness to change. The effect of the training on the MI skills was analysed with a multilevel linear regression by comparing baseline and the one-year follow-up between the interventions with usual care group. The overall effect of the consultation characteristics on the MI skills was studied in a multilevel regression analyses.

**Results:**

At one year follow up, it was demonstrated that the nurses improved on 2 of the 24 MI skills, namely, “inviting the patient to talk about behaviour change” (mean difference=0.39, *p*=0.009), and “assessing patient’s confidence in changing their lifestyle” (mean difference=0.28, *p*=0.037). Consultation time and the amount of lifestyle discussion as well as the patients’ readiness to change health behaviour was associated positively with applying MI skills.

**Conclusions:**

The maintenance of the MI skills one year after the training program was minimal. The question is whether the success of MI to change unhealthy behaviour must be doubted, whether the technique is less suitable for patients with a complex chronic disease, such as diabetes mellitus, or that nurses have problems with the acquisition and maintenance of MI skills in daily practice. Overall, performing MI skills during consultation increases, if there is more time, more lifestyle discussion, and the patients show more readiness to change.

**Trial registration:**

Current Controlled Trials ISRCTN68707773

## Background

The prevalence of type 2 diabetes is increasing mainly because of aging populations and changing lifestyles [1]. Medication, healthy diet, and physical activity can reduce blood pressure and concentrations of cholesterol and glycated hemoglobin (HbA1c), thereby lowering the risk of cardiovascular disease [2,3]. The professionals’ adherence to the type 2 diabetes guidelines on diet and physical activity is low [4-6].

In many countries, such as the Netherlands, diabetes care is largely delegated to primary care nurses. They have to make patients aware of their unhealthy lifestyles and motivate them to change. Despite nurses’ efforts to improve patients’ lifestyle, healthy behaviour change remains difficult [7,8]. A promising technique for lifestyle counselling is motivational interviewing (MI), even during brief encounters for diabetes care in general practice [9].

MI is formally defined as a patient centred, directive method for enhancing intrinsic motivation to change by exploring and resolving ambivalence [10]. Patient and professionals are jointly responsible for the treatment plan [11]. There are four general techniques of MI: (1) express empathy, (2) develop discrepancies, (3) roll with resistance, and (4) support self-efficacy (more information on MI is described in Additional file 1). Five specific methods (open questions, affirming, reflecting, summarizing, and eliciting change talk) can be useful throughout the MI. Also agenda setting, scaling questions, and assessing importance and confidence in changing lifestyle can be used as techniques to support MI [12].

Psychological interventions such as MI can be taught to nurses and incorporated in traditional diabetes settings [13-17]. However, the effect of a brief MI intervention for diabetes patients in general practice is inconclusive. Some diabetes type 2 studies have found that MI is effective in lifestyle change [13,18-21], decreasing weight [18,22], and have beneficial effects on glucose target levels, body mass index, cholesterol, and blood pressure [9,19,20,23]. Other studies showed no effect of MI on HbA1c in general practices [9,13,15], and no effect on the lifestyle, clinical parameters, quality of life and self-efficacy [13,24,25].

Information about the extent to which nurses apply MI skills and the factors that affect usage can help to understand the mixed effect of MI in routine diabetes care, but studies that looked systematically into the maintenance of the various MI skills after training are lacking [9,26,27]. The current study reports a comparison on MI skills of trained versus non-trained nurses after a one-year follow-up. The nurses participated in a cluster randomized trial in which a comprehensive program (including MI training) was tested on improving clinical parameters, lifestyle, patients’ readiness to change lifestyle, and quality of life [28]. In addition, the influence of consultation characteristics on the utilization of MI skills will be described. The consultation characteristics under study were time, the amount of nurse communication, and the amount of lifestyle discussion during consultation as well as patients’ readiness to change.

## Methods

### Study design and population

Nurses working in rural and urban general practices were recruited for a cluster randomized controlled trial in the south eastern part of the Netherlands. Randomization took place at the level of the 58 participating general practices (stratified by practice size and urbanization level) who employed a total of 65 nurses. Blinding was not possible for the nurses because the intervention group had to attend the training sessions. A complete study protocol has been described elsewhere [28].

### Intervention

Nurses in the intervention group received a comprehensive program consisting of (a) training in lifestyle counselling based on MI; (b) introduction of tools for structuring diabetes care, such as training in agenda setting, a local diabetes protocol that was discussed with them, and a social map for lifestyle support; (c) instruction for record keeping to integrate lifestyle counselling into general practice; and (d) introduction of tools to sustain improvements including an instruction chart (reminder), regular telephone follow ups with the target patients, a helpdesk that inquired proactively about the diabetes management, and a follow up meeting for the nurses (Additional file 2).

The training in MI techniques and the introduction of tools to structure diabetes care took place during the training sessions, which consisted of four half day training sessions (total 16 hours) spread over the first half year. As lifestyle education belonged already to the job of the general practice nurses, the size of the training was comparable to the study of Rubak in general practice that showed a positive effect of MI on general practitioners´ professional behaviour [29]. Nurses attended these sessions in groups of 5 to 8 outside the practice. A professional trainer provided all training sessions. The program consisted of the theory of MI, group discussions, role playing in which nurses alternately played the role of patient, nurse or observer, and an individual assignment after the training to bring the MI theory in daily practice. The record keeping and instruction chart were offered to nurses during the last training session. They received an oral and written explanation of the record keeping. It was recommended to have regular telephone follow ups that would be monthly in the first half year and probably decrease afterwards. The research team called the nurses quarterly (three times) to inquire about the progress in practicing MI, and offered a help desk that could be reached during daytime. At the request of the nurses an extra training session was planned after four months to discuss the barriers in practice and to receive feedback about their own video recording. The nurses in the control group were advised to give usual care.

### Measures and data collection

The nurses made video recordings of five type 2 diabetes consultations with different patients during the months February to May 2007 (baseline). The patients had to give consent for the recordings and its usage in the study. The recordings had to have clear sound. If video recordings failed, audio recordings were accepted. Nurses, who did not respond, were repeatedly reminded by e-mail and telephone until the program started. All nurses were asked to record again five videos of diabetes consultations after roughly a year (14 months) during the months April to September 2008. The recordings were rated with the Behaviour Change Counselling Index (BECCI) checklist [30] to evaluate the practice of the MI skills. Lane and colleagues developed the BECCI specifically to evaluate brief MI consultations. It consists of 11 items and uses a five-point rating scale (0–4) ranging from “not at all” to “a great extent”. The checklist was completed with three global items from the Motivational Interviewing Treatment Integrity instrument [31] and ten specific MI items that were addressed in the training course. These 13 items were rated on identical five-point scales as used in the BECCI. The features of the consultations that is consultation time, the amount of nurse communication, and the amount of lifestyle discussion as well as the patients’ readiness to change were identified by the judges during the rating of the videos. The judges assessed the patients´ readiness to change, by means of a predetermined scoring list. In this scoring list was described how the judges had to assess the patients’ readiness to change. It was a subjectively observation that could be expressed on a five-point rating scale (0–4) ranging from “not at all” to “a great extent”. The nurses’ demographic characteristics and data about their experiences as nurses or with MI were collected in self-reported questionnaires at baseline.

### Rating consultations

The first author (RJ) trained two judges (CS and NV) to rate the recorded consultations. They examined the recordings twice, made notes, and gave their judgments. The judges were independent and were blinded for allocation of the nurses to the intervention or control group.

### Ethical considerations

The Medical Ethics Committee of the Radboud University Nijmegen approved this study. Nurses received an invitation letter with information about the study, the possibility of withdrawing at any time, and the guarantee of confidentiality. Consultations were only recorded after the patients were informed about the aim of the study and had given their permission.

### Data analysis

Means with ranges or percentages were used to describe the nurse characteristics. The intra-class correlation coefficient (ICC) was calculated to determine the agreement of the two judges. For the items on the BECCI checklist as well as the additional items about MI the Cronbach’s alpha was used. The MI skills are expressed by means and standard deviations based on the items of the BECCI checklists and the additional items at baseline and at one-year follow-up. To compare the intervention group with usual care on the different MI skills a multilevel linear regression analysis (three levels: nurse, consultation, and measurement) was performed. Separate models were estimated for the BECCI list and the additional items; both models were adjusted for nurse characteristics that differed significantly between the intervention and usual care groups at baseline. Differences were considered significant if *p* < 0.05. To establish, overall, which consultation characteristics might influence the utilization of MI skills, the one-year follow-up data were analysed in a multilevel regression (top down procedure); again these analyses were performed separately for the BECCI items and the additional MI items. The least and non-significant components were deleted step by step, separately for both models. Intervention effects were examined in the most reduced models with a significance of 0.05 as the cut-off point. SPSS for Windows was used for the statistical analyses.

## Results

### Study population

**Figure 1:**
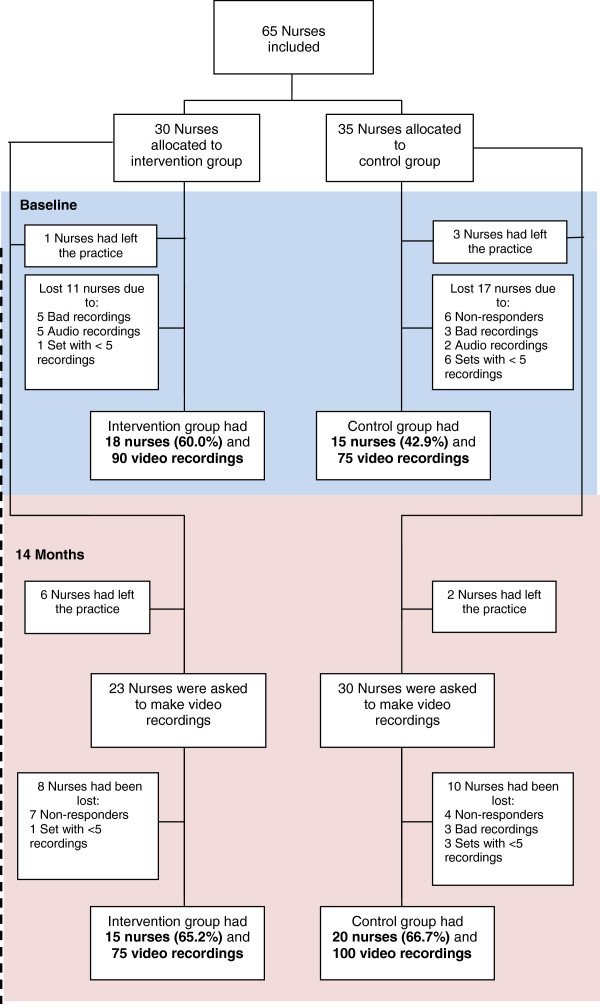
Flow diagram showing numbers of participants.

Figure 1 presents the numbers of general practices and nurses in this trial. Sixty-five nurses participated in the study; 30 nurses were trained [28], while 35 nurses in the control group were invited to take the training course after the intervention. Sixty percent of the intervention group nurses and 43% of the control group nurses supplied five usable baseline video recordings; 65% of intervention group nurses and 67% of the control group nurses supplied five recordings at one-year follow-up.

The control group had significantly more experience as practice assistants than the intervention group (Table 1). A practice assistant in the Netherlands is someone who assists a doctor and works predominantly as a receptionist and administrative assistant [32]. Since 1999, practice assistants can follow a 2 year training to become a primary care nurse. The non-responders did not differ significantly from the responders with regard to sex, age, background, experience as practice assistants, and experience with diabetes consultations.

**Table 1:** Nurses’ characteristics at baseline

	**Intervention group**^**a**^**Control group**^**a**^	
**Nurses**	20	23
Percentage of male nurses	10.0	4.3
Mean age in years (range)	41.6 (27–57)	43.7 (31–57)
Percentage of nurses who were formerly practice assistants	45.0	60.9
Mean years of experience as a practice assistant (range) *	**4.4 (0–16)**	**9.7 (0–28)**
Mean years of experience with diabetes consultations (range)	3.6 (0–10)	4.1 (1–8)
Percentage of nurses who had training in motivational interviewing besides the MILD training	30.4	40.7
Percentage of nurses who engaged in other motivational interviewing activities besides the MILD training	43.5	40.7

*p < 0.05.

^a^ Nurses who participated at baseline and/or 14 months later.

MILD, Motivational interviewing to change the lifestyle of patients with type 2 diabetes.

### Improvement of MI skills

The audio recordings (n=35) were difficult to rate, because information was missing or more difficult to interpret. Therefore, the audio recordings were disregarded and only video recordings were allowed for the one-year follow-up. Table 2 shows that nurses only showed a significant improvement in 2 of the MI skills at one-year follow-up compared to baseline. The mean scores for most items were below point two on the 5-point scales. The BECCI checklist gave an ICC of 0.79 for the two judges and a Cronbach’s alpha of 0.88 that can be judged as “good”. A small, but significant, improvement in the intervention group versus the control group was seen for just one item: “nurse invites the patient to talk about behaviour change” (Table 2). The ICC for the two judges on the 13 additional MI items was 0.67, and these items had Cronbach’s alpha of 0.63 that can be judged as “moderately”. There was a small, significant improvement in the intervention group compared to the control group in the score for “nurse assesses patient’s confidence in changing their lifestyle” (Table 2).

**Table 2:** Scores on the behaviour change counselling index checklist and additional questions

	**Baseline**		**One-year follow-up**		**Effects**			
Range 0 (not at all) – 4 (a great extent)	Intervention	Control	Intervention	Control	Intervention	Control	Difference in change between groups	*p* value
**Motivational interviewing: mean scores (s.d.) on the BECCI**	1.53 (0.47)	1.49 (0.45)	1.63 (0.65)	1.42 (0.52)	0.07 (0.09)	−0.08 (0.09)	0.15 (0.12)	0.237
1. Nurse invites the patient to talk about behaviour change	1.43 (0.77)	1.67 (0.69)	1.50 (0.78)	1.36 (0.77)	0.13 (0.11)	−0.26 (0.11)*	0.39 (0.15)*	0.009*
2. Nurse demonstrates sensitivity to talking about other issues	2.77 (0.68)	2.78 (0.65)	2.92 (0.64)	2.56 (0.62)	0.19 (0.13)	0.05 (0.13)	0.14 (0.18)	0.440
3. Nurse encourages patient to talk about current behaviour	2.00 (0.93)	1.93 (0.86)	1.89 (1.04)	1.72 (0.96)	−0.20 (0.14)	−0.21 (0.14)	0.01 (0.19)	0.941
4. Nurse encourages patient to talk about change	1.62 (0.92)	1.61 (0.85)	1.76 (1.07)	1.50 (0.82)	0.10 (0.14)	−0.09 (0.14)	0.19 (0.20)	0.343
5. Nurse asks questions to find out how patient thinks and feels about topic	0.93 (1.04)	0.85 (1.00)	1.37 (1.24)	0.85 (1.00)	0.41 (0.18)*	−0.00 (0.18)	0.41 (0.25)	0.110
6. Nurse uses empathic listening statements when the patient talks about the topic	2.24 (0.64)	2.11 (0.61)	2.25 (0.95)	2.11 (0.71)	−0.01 (0.11)	0.02 (0.11)	−0.03 (0.15)	0.846
7. Nurse uses summaries to bring together what the patient says about the topic	0.20 (0.45)	0.16 (0.44)	0.46 (0.65)	0.24 (0.47)	0.19 (0.07)*	0.11 (0.07)	0.08 (0.09)	0.395
8. Nurse acknowledges challenges about behaviour change that the patient faces	2.22 (0.80)	2.12 (0.72)	2.08 (0.95)	1.96 (1.10)	0.03 (0.13)	−0.07 (0.12)	0.10 (0.18)	0.562
9. When nurse provides information, she is sensitive to the patient’s concerns and understanding	1.72 (0.60)	1.77 (0.94)	1.59(0.60)	1.40 (0.63)	−0.07 (0.08)	−0.17 (0.08)*	0.10 (0.11)	0.378
10. Nurse actively conveys respect for patient choice about behaviour change	1.82 (0.75)	1.93 (0.72)	1.82 (1.04)	1.85 (0.92)	0.03 (0.14)	−0.05 (0.13)	0.08 (0.19)	0.679
11. Nurse and patient exchange ideas about how the patient could change current behaviour	1.29 (0.98)	1.45 (1.05)	1.27 (1.15)	1.39 (1.12)	−0.07 (0.14)	−0.07 (0.14)	−0.00 (0.20)	0.984
**Motivational interviewing: mean scores (s.d.) on additional questionnaire**	1.21 (0.25)	1.16 (0.23)	1.42 (0.36)	1.24 (0.23)	0.19 (0.05)*	0.08 (0.05)	0.11 (0.07)	0.133
1. Global score: empathy	2.16 (0.36)	2.05 (0.41)	2.37 (0.54)	2.20 (0.47)	0.20 (0.08)*	0.15 (0.08)	0.05 (0.11)	0.667
2. Global score: spirit	1.92 (0.37)	1.94 (0.39)	2.04 (0.51)	1.91 (0.35)	0.09 (0.06)	−0.03 (0.06)	0.12 (0.08)	0.146
3. Global score: structure in consultation	2.86 (0.36)	2.89 (0.35)	2.93 (0.26)	2.93 (0.26)	0.05 (0.06)	0.06 (0.06)	0.01 (0.07)	0.902
4. Nurse applies agenda setting and gives structure to the consultation	1.06 (0.27)	1.13 (0.47)	1.11 (0.28)	1.17 (0.37)	0.05 (0.06)	0.05 (0.06)	−0.00 (0.08)	0.955
5. Nurse assesses patient’s importance of changing their undesirable lifestyle	0.11 (0.36)	0.15 (0.41)	0.33 (0.61)	0.18 (0.45)	0.13 (0.09)	0.01 (0.09)	0.13 (0.13)	0.314
6. Nurse assesses patient’s confidence in changing lifestyle	0.16 (0.42)	0.27 (0.51)	0.62 (0.78)	0.35 (0.67)	0.33 (0.09)*	0.05 (0.09)	0.28 (0.13)*	0.037*
7. Nurse draws up concrete and feasible goals with the patient	0.69 (0.85)	0.73 (0.90)	1.11 (0.99)	0.92 (0.92)	0.38 (0.14)*	0.19 (0.14)	0.19 (0.20)	0.360
8. Nurse rolls with resistance, is flexible, and avoids discussion	1.85 (0.80)	1.90 (0.88)	2.03 (0.93)	2.07 (0.72)	0.26 (0.10)*	0.15 (0.10)	0.11 (0.14)	0.415
9. Nurse supports and reinforces the self-efficacy of the patient	0.56 (0.96)	0.40 (0.88)	1.11 (1.28)	0.64 (0.96)	0.56 (0.15)*	0.24 (0.15)	0.32 (0.21)	0.126
10. Nurse highlights and helps resolve discrepancy between present behaviour and important personal goals	0.00 (0.00)	0.05 (0.21)	0.14 (0.35)	0.09 (0.28)	0.14 (0.04)*	0.04 (0.04)	0.10 (0.05)	0.054
11. Nurse asks open questions instead of closed questions as often as possible	1.71 (1.52)	1.30 (1.50)	1.55 (1.55)	1.21 (1.51)	−0.24 (0.22)	−0.08 (0.22)	−0.16 (0.32)	0.625
12. Nurse applies reflections	2.14 (0.35)	2.04 (0.36)	2.17 (0.41)	2.08 (0.35)	0.01 (0.05)	0.04 (0.05)	−0.04 (0.08)	0.636
13. Nurse is sitting behind the chair	0.49 (0.58)	0.31 (0.52)	0.94 (0.95)	0.40 (0.63)	0.43 (0.17)*	0.10 (0.17)	0.33 (0.24)	0.177

*p < 0.05 was considered significant.

^a^ BECCI, Behaviour Change Counselling Index.

The values were adjusted for experience as a practice assistant.

### Consultation characteristics

The mean duration of consultation differed significantly between intervention (21.8 minutes) and control group (17.6 minutes). Table 3 shows that nurses used significantly more MI skills measured by the BECCI if the consultation took more time (B-estimate= 0.13; s.d.= 0.05), and when more time was spent discussing lifestyle factors (B-estimate = 8.97; s.d.= 0.71). The time lifestyle was discussed was also positively associated with the additional MI items (B-estimate= 4.28; s.d.= 0.53) as well as the patients readiness to change (B-estimate= 1.41; s.d.= 0.58).

**Table 3:** Regression between consultation characteristics and the extent to which nurses applied motivational interviewing

	**Motivational interviewing BECCI**			**Motivational interviewing additional questions**		
**Consultations** (*n* = 175)	*B estimate*	*s.d.*	*p-value*	*B estimate*	*s.d.*	*p-value*
Consultation time in minutes	0.13	0.05	0.011*	-	-	-
Amount of nurse talk during consultation	-	-	-	-	-	-
Time given to lifestyle during consultation	8.98	0.71	0.000*	4.28	0.53	0.000*
Patients’ readiness to change	-	-	-	1.41	0.58	0.015*

*p < 0.05.

BECCI; Behaviour Change Counselling Index.

## Discussion

The MI training embedded in a comprehensive program to improve routine diabetes care in general practice had a minimal impact upon lifestyle counseling practice of MI skills when assessed at one- year follow-up. The comparison of video consultations in a cluster randomized controlled trial showed that two of the 24 MI skills improved, that is “the invitation to talk about behavioural change” and “the assessment of patients’ confidence to change”. In general, it can be stated that nurses showed more MI skills if the consultations took more time and when more lifestyle issues were discussed. The observed patients’ readiness to change was also positively related to the degree of MI skills expressed.

### Strengths and limitations of the study

A strength of the study is the RCT design, and the number of videos that could be rated (n=340).

Clinical trials on MI in diabetes care seldom include a fidelity check of the actual use of MI skills [19,29,33]. Rubak et al. (2006) described that general practitioners changed their behaviour in daily practice after an MI course [29]. However, this study used self-reported data, which tend to be less reliable than objective measures such as observations [34]. Miller et al. (2004) assessed MI skills after training with audio taped samples that lack non-verbal information [35]. They argued that appropriate assessment of MI practice is necessary in studies on MI in order to explore the effects of true MI practice, and that direct monitoring of practice is the gold standard, since self-report of the MI practitioners are unreliable [35]. Such direct monitoring in this study was performed by means of tape recordings of counseling sessions.

Another strength of the study is the high agreement among the judges. The checklists used to rate MI skills (BECCI, and some additional questions) probably supported the rating process well. A possible limitation of the study is a bias due to the self-selection of the video recordings of the consultations. Nurses told us that it was very difficult to arrange a good camera setting. They often had to borrow a camera. Based on this feedback, it was assumed that being highly selective of taped consultations was not feasible. This endorses the results that the intervention had a minimal impact upon MI skill in routine diabetes care.

### MI skills at one-year follow-up

It is difficult to compare the study results with other studies, because few studies measured the effect of training on nurses’ MI skills in diabetes care and evaluated it in such detail with recordings of diabetes consultations. At one-year follow-up, it was demonstrated that the nurses improved minimally on their MI skills. This is in line with previous findings suggesting that MI skills are not easily applicable in daily practice [17,26]. Others, showed that health care practitioners who wished to learn MI were able to acquire MI skills up to at least beginning proficiency and transfer these skills to a real life clinical setting [14], but in this study it is not known whether the practitioners continued to use MI in routine practice after the study period. Miller et al. (2004) reported, based on audio tapes, that the intervention group showed greater gains in MI skills than the control group, but MI performance diminished without further training support [35]. This phenomenon could also have affected the presented study results.

### Lacking lifestyle counselling skills

Nurses often state that skills for lifestyle counselling are lacking [36,37]. In many countries, such as the Netherlands, diabetes care is largely delegated to primary care nurses. Nurses are trained in a three to four years curriculum (middle or higher education) and afterwards they can specialize in primary care following a one or two years curriculum. In the curriculum, interviewing techniques are addressed but not specific to MI. Consequently, the nurses who participated were trained in MI, such as suggested by Rubak et al. [29], but they showed little change in MI skills when assessed at one-year follow-up. A review supported the idea that our number of MI training sessions was sufficient [38]. Other studies suggest that training alone is not enough for acquiring MI skills. Ongoing coaching/ feedback, written material and supervision are also essential [14,16,17,39]. Continued support on the job seems to be needed [40] in which MI skills can be practiced and evaluated during daily routine over a longer period of time. Moreover, it is important that health care providers are supported by their supervisor and colleagues in performing MI [27].

### Separate MI sessions

The association of time and time spent on lifestyle discussion with MI skills has been concluded by others as well [17] and it fits into the debate that separate MI sessions are successful [22,41]. In the Netherlands, nurses see patients with type 2 diabetes four times a year for 15 to 20 minutes. In these diabetes consultations glucose level, blood pressure, and weight must be measured, information about the effect of the medication is updated, the nurse attempts to educate the patient about diabetes and the relation of diabetes complications to diet, physical activity, and smoking behaviour. The nurses in the intervention group took on average 21.8 minutes consultation time which was 4 minutes more than in the control group. But, possible this time is not enough for performing successful lifestyle counselling. Making lifestyle counselling part of diabetes care seems to require more time that probably is easier to realize in a separate MI session.

### Diabetes care and MI

MI was originally developed for substance abuse [10] which requires a single behavioural change. MI may be less effective for multiple behavioural changes needed in a complex chronic illness such as diabetes mellitus. Although, some studies showed an effect of MI on diabetes care [9,19,21,22].

However, these studies seldom observed MI skills in practice. Therefore, it is unclear if the effect can be attributed to MI skills or to the intervention in general. Our comprehensive diabetes program, including the training, had no significant effect on HbA1c, blood pressure and cholesterol level nor on reported aspects of lifestyle or quality of life [42]. Since, the MI skills showed minimal improvement at one-year follow-up, the attribution problem cannot be solved yet. It is possible that more training is needed or that MI only can be effective in certain groups of people. A review showed that for type 1 diabetes psychological treatments could improve glycaemic controls, but only in children and adolescents [43]. More studies are needed that probably will sketch a nuanced picture.

## Conclusion

The utilization of MI skills of primary care nurses showed minimal improvement after MI training embedded in a comprehensive program to improve routine diabetes care. It is unclear if the success of MI as such should be questioned, whether the technique is less appropriate in routine care for patients with more complex lifestyle conditions, or that nurses have problems with the acquisition and maintenance of MI skills in daily practice.

## Competing interests

The authors declare that there is no conflict of interest.

## Author’s contributions

RJ, the main investigator, participated in the development and implementations of the methodology, the analysis and interpretation of results and was responsible for preparing the manuscript. JB, the project leader, involved in all aspects of the study. ML participated in revising the manuscript and EK performed the statistical analyses. TvdW, GE, and RG participated in the development of the study, interpretation of the results and revising the manuscript. All authors read and approved the final version of the manuscript.

## Pre-publication history

The pre-publication history for this paper can be accessed here:

http://www.biomedcentral.com/1471-2296/14/44/prepub

## Supplementary Material

Additional file 1 **Description of motivational interviewing.** A promising technique for lifestyle counselling is motivational interviewing (MI). In this Additional file more information about MI is described, such as the definition of MI, and the four general principles and five specific methods of MI. Click here for file

Additional file 2 **The several interventions of the comprehensive diabetes program.** In this additional file, the several interventions of the comprehensive diabetes program is described. The comprehensive program consisting of (a) training in lifestyle counselling based on MI; (b) introduction of tools for structuring diabetes care; (c) instruction for record keeping to integrate lifestyle counselling into general practice; and (d) introduction of tools to sustain improvements. Click here for file
