# Supplementary material for: Minimal improvement of nurses’ motivational interviewing skills in routine diabetes care one year after training: a cluster randomized trial
Source: BMC Fam Pract. 2013 Mar 28;14:44. doi: 10.1186/1471-2296-14-44 (PMC3637576; doi:10.1186/1471-2296-14-44)
Supplement: Additional file 2 — The several interventions of the comprehensive diabetes program. In this additional file, the several interventions of the comprehensive diabetes program is described. The comprehensive program consisting of (a) training in lifestyle counselling based on MI; (b) introduction of tools for structuring diabetes care; (c) instruction for record keeping to integrate lifestyle counselling into general practice; and (d) introduction of tools to sustain improvements. [file 1471-2296-14-44-S2.doc]

**Additional file 2.** The several interventions of the comprehensive diabetes program

***Training for motivational interviewing*** (4 half days, spread equally over 6 months)

1. **Training** primary care nurses in the principles of **motivational interviewing (MI)** in order to encourage patients with diabetes to adhere to lifestyle guidelines. The following components were discussed:

- *Building motivation for change: importance and confidence*
- *Asking open questions, listening reflectively, affirming, summarizing, and eliciting change*
- *Expressing empathy, developing discretion, rolling with resistance, and supporting self-efficacy.*

***Structured diabetes care***

1. Training in **agenda setting** to make consultations more structured and to draw up concrete appointments
2. Tailoring a **diabetes protocol** to the local setting
3. Introducing a **social map** for lifestyle change to primary care nurses. The map is an overview of all available organizations and their treatment programs to help patients choose, for example, the right sport school or physiotherapist.

***Lifestyle counselling embedded in usual care***

1. **Record keeping** of consultation data and behavioural change of the patients, which primary care nurses must do.

***Maintaining motivational interviewing***

*Reminder*

1. An **instruction chart** with counselling techniques, as a reminder for nurses to maintain the MI techniques to help patients change.

*Follow–up*

1. Recommendations for **regular telephone follow-ups** for diabetes patients, which will be monthly in the 1st half year and then will probably decrease
2. A **help-desk**: the research team will call the primary care nurse three times to inquire about their development of health counselling, and nurses can call the research team for information
3. A **follow-up meeting** to receive feedback about their own video recording.
